# 4-[(1*H*-Benzotriazol-1-yl)meth­yl]benzo­nitrile

**DOI:** 10.1107/S1600536808010969

**Published:** 2008-05-03

**Authors:** Wen-Xiang Wang, Hong Zhao

**Affiliations:** a Ordered Matter Science Research Center, College of Chemistry and Chemical Engineering, Southeast University, Nanjing 210096, People’s Republic of China

## Abstract

In the mol­ecule of the title compound, C_14_H_10_N_4_, which was prepared by reaction of benzotriazole with 4-(bromo­meth­yl)benzonitrile in alkaline solution, the dihedral angle between the benzotriazole and benzene ring systems is 69.03 (6)°.

## Related literature

For the application of benzotriazole compounds in industry, see: Pillard *et al.* (2001 ▶); Kopanska *et al.* (2004 ▶); Gruden *et al.* (2001 ▶). For the structure of a related compound, see: Selvanayagam *et al.* (2002 ▶).
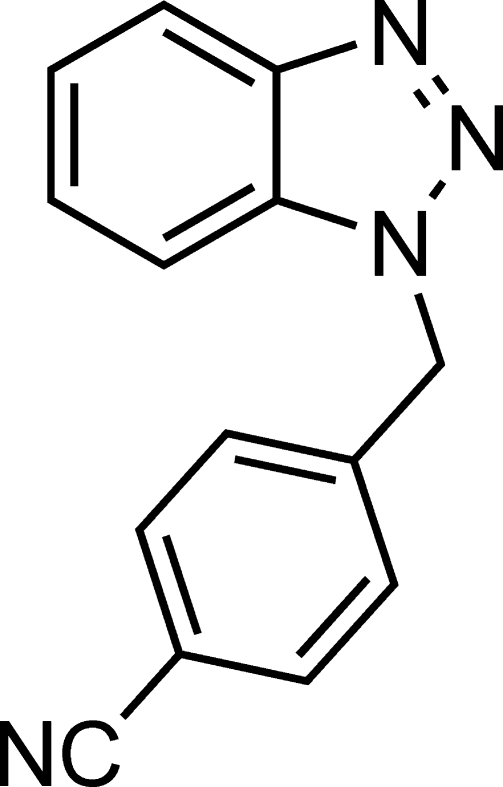


## Experimental

### 

#### Crystal data

C_14_H_10_N_4_
*M* *_r_* = 234.26
Monoclinic, 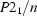

*a* = 8.1912 (13) Å
*b* = 19.0520 (9) Å
*c* = 8.6610 (6) Å
β = 118.0390 (10)°
*V* = 1193.0 (2) Å^3^
*Z* = 4
Mo *K*α radiation
μ = 0.08 mm^−1^
*T* = 293 (2) K
0.50 × 0.40 × 0.40 mm

#### Data collection

Rigaku Mercury2 diffractometer
Absorption correction: multi-scan (*CrystalClear*; Rigaku, 2005 ▶) *T*_min_ = 0.960, *T*_max_ = 0.969
11866 measured reflections
2715 independent reflections
1903 reflections with *I* > 2σ(*I*)
*R*_int_ = 0.047

#### Refinement

*R*[*F* ^2^ > 2σ(*F* ^2^)] = 0.067
*wR*(*F* ^2^) = 0.182
*S* = 1.09
2715 reflections
163 parameters
H-atom parameters constrained
Δρ_max_ = 0.34 e Å^−3^
Δρ_min_ = −0.17 e Å^−3^

### 

Data collection: *CrystalClear* (Rigaku, 2005 ▶); cell refinement: *CrystalClear*; data reduction: *CrystalClear*; program(s) used to solve structure: *SHELXS97* (Sheldrick, 2008 ▶); program(s) used to refine structure: *SHELXL97* (Sheldrick, 2008 ▶); molecular graphics: *SHELXTL* (Sheldrick, 2008 ▶); software used to prepare material for publication: *SHELXTL*.

## Supplementary Material

Crystal structure: contains datablocks I, global. DOI: 10.1107/S1600536808010969/rz2206sup1.cif

Structure factors: contains datablocks I. DOI: 10.1107/S1600536808010969/rz2206Isup2.hkl

Additional supplementary materials:  crystallographic information; 3D view; checkCIF report

## Figures and Tables

**Fig. 1.**
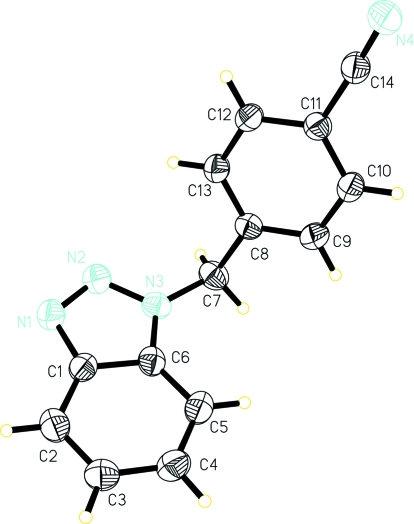
The molecular structure of the title compound with the atom-numbering scheme. Displacement ellipsoids were drawn at the 30% probability level.

## supplementary crystallographic information

### Comment

Benzotriazole and its derivatives comprise an important class of corrosion inhibitors, typically used as trace additives in industrial chemical mixtures, such as coolants, cutting fluids and hydraulic fluid (Pillard *et al.*, 2001). These derivatives are also used as inhibitors of Acanthamoeba castellanii (Kopanska *et al.*, 2004) and are responsible for toxicity to bacteria (Gruden *et al.*, 2001). In this paper the crystal structure of the title compound is reported.

In the title compound, bond lengths and angles observed in the benzotriazole ring system are comparable with those reported in other benzotriazole compounds (Selvanayagam *et al.*, 2002). The dihedral angle between benzotriazole and benzene rings is 69.03 (6)°. The crystal structure is stabilized only by van der Waals contacts.

### Experimental

A mixture of benzotriazole (0.01 mol) and KOH (0.56 g) in methanol (20 ml) was stirred for 10 min. 4-(Bromomethyl)benzonitrile (0.01 mol) was then added and the solution refluxed for 24 h. After completion of the reaction, the reaction mixture was evaporated under vacuum. Yellow crystals of the title compound suitable for X-ray diffraction were obtained by slow evaporation of an ethanol solution after 3 days.

### Refinement

All H atoms were calculated geometrically and were refined using the riding-model approximation, with C—H =0.93-0.87 Å and with *U*_iso_(H) = 1.2*U*_eq_ (C).

### Crystal data

**Table:**

C_14_H_10_N_4_	*F*_000_ = 488
*M**_r_* = 234.26	*D*_x_ = 1.304 Mg m^−^^3^
Monoclinic, *P*2_1_/*n*	Mo *K*α radiation λ = 0.71073 Å
Hall symbol: -P 2yn	Cell parameters from 2264 reflections
*a* = 8.1912 (13) Å	θ = 3.0–27.4º
*b* = 19.0520 (9) Å	µ = 0.08 mm^−^^1^
*c* = 8.6610 (6) Å	*T* = 293 (2) K
β = 118.0390 (10)º	Block, yellow
*V* = 1193.0 (2) Å^3^	0.50 × 0.40 × 0.40 mm
*Z* = 4	

### Data collection

**Table:**

Rigaku Mercury2 diffractometer	2715 independent reflections
Radiation source: fine-focus sealed tube	1903 reflections with *I* > 2σ(*I*)
Monochromator: graphite	*R*_int_ = 0.047
Detector resolution: 13.6612 pixels mm^-1^	θ_max_ = 27.5º
*T* = 293(2) K	θ_min_ = 3.0º
CCD Profile fitting scans	*h* = −10→10
Absorption correction: multi-scan(CrystalClear; Rigaku, 2005)	*k* = −24→24
*T*_min_ = 0.960, *T*_max_ = 0.969	*l* = −11→11
11866 measured reflections	

### Refinement

**Table:**

Refinement on *F*^2^	Secondary atom site location: difference Fourier map
Least-squares matrix: full	Hydrogen site location: inferred from neighbouring sites
*R*[*F*^2^ > 2σ(*F*^2^)] = 0.067	H-atom parameters constrained
*wR*(*F*^2^) = 0.182	*w* = 1/[σ^2^(*F*_o_^2^) + (0.0763*P*)^2^ + 0.2728*P*] where *P* = (*F*_o_^2^ + 2*F*_c_^2^)/3
*S* = 1.09	(Δ/σ)_max_ = 0.002
2715 reflections	Δρ_max_ = 0.34 e Å^−^^3^
163 parameters	Δρ_min_ = −0.17 e Å^−^^3^
Primary atom site location: structure-invariant direct methods	Extinction correction: none

### Special details

**Table:**

Geometry. All e.s.d.'s (except the e.s.d. in the dihedral angle between two l.s. planes) are estimated using the full covariance matrix. The cell e.s.d.'s are taken into account individually in the estimation of e.s.d.'s in distances, angles and torsion angles; correlations between e.s.d.'s in cell parameters are only used when they are defined by crystal symmetry. An approximate (isotropic) treatment of cell e.s.d.'s is used for estimating e.s.d.'s involving l.s. planes.
Refinement. Refinement of *F*^2^ against ALL reflections. The weighted *R*-factor *wR* and goodness of fit *S* are based on *F*^2^, conventional *R*-factors *R* are based on *F*, with *F* set to zero for negative *F*^2^. The threshold expression of *F*^2^ > σ(*F*^2^) is used only for calculating *R*-factors(gt) *etc*. and is not relevant to the choice of reflections for refinement. *R*-factors based on *F*^2^ are statistically about twice as large as those based on *F*, and *R*- factors based on ALL data will be even larger.

### Fractional atomic coordinates and isotropic or equivalent isotropic displacement parameters (Å^2^)

**Table:**

	*x*	*y*	*z*	*U*_iso_*/*U*_eq_	
C1	0.7006 (4)	0.10232 (14)	0.9067 (4)	0.0662 (7)	
H1A	0.6891	0.1496	0.8769	0.079*	
C2	0.8443 (4)	0.07658 (15)	1.0621 (4)	0.0714 (7)	
H2B	0.9320	0.1081	1.1380	0.086*	
C3	0.8630 (4)	0.00549 (15)	1.1096 (4)	0.0706 (7)	
C4	0.7387 (4)	−0.04391 (14)	1.0033 (4)	0.0674 (7)	
H4A	0.7493	−0.0911	1.0344	0.081*	
C5	0.5915 (3)	−0.01855 (12)	0.8418 (3)	0.0554 (6)	
C6	0.5771 (3)	0.05233 (12)	0.8006 (3)	0.0546 (6)	
C7	0.3403 (4)	0.11817 (13)	0.5272 (3)	0.0631 (6)	
H7A	0.2790	0.1033	0.4058	0.076*	
H7B	0.4372	0.1510	0.5420	0.076*	
C8	0.2010 (3)	0.15545 (12)	0.5681 (3)	0.0513 (5)	
C9	0.2298 (3)	0.22412 (12)	0.6302 (3)	0.0626 (6)	
H9A	0.3379	0.2474	0.6504	0.075*	
C10	0.0989 (3)	0.25833 (13)	0.6624 (3)	0.0616 (6)	
H10A	0.1182	0.3044	0.7026	0.074*	
C11	−0.0614 (3)	0.22289 (11)	0.6339 (3)	0.0515 (5)	
C12	−0.0901 (3)	0.15394 (12)	0.5744 (3)	0.0583 (6)	
H12A	−0.1967	0.1302	0.5566	0.070*	
C13	0.0408 (3)	0.12081 (12)	0.5417 (3)	0.0574 (6)	
H13A	0.0212	0.0747	0.5015	0.069*	
C14	−0.2037 (4)	0.25735 (12)	0.6617 (3)	0.0621 (6)	
N1	0.4254 (3)	0.05596 (10)	0.6402 (3)	0.0574 (5)	
N2	0.3499 (3)	−0.00893 (11)	0.5862 (3)	0.0684 (6)	
N3	0.4492 (3)	−0.05515 (11)	0.7082 (3)	0.0688 (6)	
N4	−0.3207 (3)	0.28251 (13)	0.6795 (4)	0.0841 (8)	
H3B	0.9617	−0.0085	1.2151	0.101*	

### Atomic displacement parameters (Å^2^)

**Table:**

	*U*^11^	*U*^22^	*U*^33^	*U*^12^	*U*^13^	*U*^23^
C1	0.0730 (16)	0.0558 (14)	0.0776 (18)	−0.0081 (12)	0.0417 (14)	−0.0063 (12)
C2	0.0659 (16)	0.0753 (18)	0.0646 (17)	−0.0119 (13)	0.0238 (13)	−0.0063 (13)
C3	0.0653 (16)	0.0776 (18)	0.0631 (16)	−0.0004 (13)	0.0253 (13)	−0.0002 (13)
C4	0.0726 (17)	0.0598 (15)	0.0774 (18)	0.0070 (12)	0.0417 (15)	0.0072 (13)
C5	0.0539 (13)	0.0533 (13)	0.0677 (15)	−0.0040 (10)	0.0358 (12)	−0.0103 (11)
C6	0.0604 (14)	0.0511 (12)	0.0641 (14)	0.0022 (10)	0.0390 (12)	−0.0023 (10)
C7	0.0645 (15)	0.0679 (15)	0.0641 (15)	0.0104 (12)	0.0361 (12)	0.0075 (12)
C8	0.0492 (12)	0.0554 (13)	0.0497 (12)	0.0039 (9)	0.0235 (10)	0.0039 (10)
C9	0.0508 (13)	0.0606 (15)	0.0791 (17)	−0.0085 (10)	0.0327 (12)	−0.0035 (12)
C10	0.0604 (14)	0.0509 (13)	0.0748 (16)	−0.0079 (11)	0.0328 (12)	−0.0098 (11)
C11	0.0509 (12)	0.0520 (12)	0.0526 (13)	0.0003 (9)	0.0252 (10)	−0.0016 (10)
C12	0.0527 (13)	0.0531 (13)	0.0713 (15)	−0.0084 (10)	0.0310 (12)	−0.0083 (11)
C13	0.0583 (14)	0.0478 (12)	0.0672 (15)	−0.0029 (10)	0.0303 (12)	−0.0084 (10)
C14	0.0659 (15)	0.0530 (13)	0.0762 (16)	−0.0044 (11)	0.0408 (13)	−0.0065 (12)
N1	0.0533 (11)	0.0651 (12)	0.0564 (12)	0.0047 (9)	0.0279 (9)	−0.0029 (9)
N2	0.0658 (13)	0.0603 (13)	0.0782 (15)	0.0012 (10)	0.0330 (11)	−0.0047 (11)
N3	0.0681 (13)	0.0547 (12)	0.0851 (15)	−0.0019 (10)	0.0373 (12)	−0.0069 (11)
N4	0.0786 (16)	0.0733 (15)	0.119 (2)	−0.0010 (12)	0.0622 (16)	−0.0153 (14)

### Geometric parameters (Å, °)

**Table:**

C1—C6	1.379 (3)	C7—H7B	0.9700
C1—C2	1.396 (4)	C8—C13	1.388 (3)
C1—H1A	0.9301	C8—C9	1.392 (3)
C2—C3	1.403 (4)	C9—C10	1.391 (3)
C2—H2B	0.9300	C9—H9A	0.9299
C3—C4	1.374 (4)	C10—C11	1.391 (3)
C3—H3B	0.9299	C10—H10A	0.9300
C4—C5	1.435 (4)	C11—C12	1.390 (3)
C4—H4A	0.9300	C11—C14	1.453 (3)
C5—N3	1.384 (3)	C12—C13	1.383 (3)
C5—C6	1.387 (3)	C12—H12A	0.9299
C6—N1	1.362 (3)	C13—H13A	0.9300
C7—N1	1.486 (3)	C14—N4	1.145 (3)
C7—C8	1.521 (3)	N1—N2	1.363 (3)
C7—H7A	0.9700	N2—N3	1.321 (3)
			
C6—C1—C2	114.9 (2)	C13—C8—C9	119.1 (2)
C6—C1—H1A	122.5	C13—C8—C7	119.6 (2)
C2—C1—H1A	122.5	C9—C8—C7	121.3 (2)
C1—C2—C3	123.2 (2)	C10—C9—C8	120.7 (2)
C1—C2—H2B	118.4	C10—C9—H9A	119.6
C3—C2—H2B	118.4	C8—C9—H9A	119.6
C4—C3—C2	121.4 (2)	C11—C10—C9	119.3 (2)
C4—C3—H3B	119.4	C11—C10—H10A	120.4
C2—C3—H3B	119.3	C9—C10—H10A	120.4
C3—C4—C5	116.2 (2)	C12—C11—C10	120.4 (2)
C3—C4—H4A	121.9	C12—C11—C14	118.6 (2)
C5—C4—H4A	121.9	C10—C11—C14	121.0 (2)
N3—C5—C6	109.8 (2)	C13—C12—C11	119.7 (2)
N3—C5—C4	129.6 (2)	C13—C12—H12A	120.2
C6—C5—C4	120.6 (2)	C11—C12—H12A	120.2
N1—C6—C1	132.6 (2)	C12—C13—C8	120.8 (2)
N1—C6—C5	103.8 (2)	C12—C13—H13A	119.6
C1—C6—C5	123.6 (2)	C8—C13—H13A	119.6
N1—C7—C8	112.97 (18)	N4—C14—C11	177.3 (3)
N1—C7—H7A	109.0	C6—N1—N2	110.69 (19)
C8—C7—H7A	109.0	C6—N1—C7	129.3 (2)
N1—C7—H7B	109.0	N2—N1—C7	120.0 (2)
C8—C7—H7B	109.0	N3—N2—N1	108.7 (2)
H7A—C7—H7B	107.8	N2—N3—C5	107.0 (2)
